# Molecular detection of vector-borne pathogens in dogs and cats from Qatar

**DOI:** 10.1186/s13071-017-2237-y

**Published:** 2017-06-20

**Authors:** Ana Margarida Alho, Clara Lima, Maria Stefania Latrofa, Vito Colella, Silvia Ravagnan, Gioia Capelli, Luís Madeira de Carvalho, Luís Cardoso, Domenico Otranto

**Affiliations:** 1 CIISA, Faculdade de Medicina Veterinária, Universidade de Lisboa, Lisbon, Portugal; 2 Hospital Parkview Pet Center – Veterinary Clinic, Doha, Qatar; 3 Dipartimento di Medicina Veterinaria, Università degli Studi di Bari, Bari, Italy; 4 Istituto Zooprofilattico Sperimentale delle Venezie, Legnaro, Padova, Italy; 5 Department of Veterinary Sciences, School of Agrarian and Veterinary Sciences, University of Trás-os-Montes e Alto Douro (UTAD), Vila Real, Portugal

**Keywords:** *Anaplasma platys*, *Babesia felis*, *Babesia gibsoni*, *Babesia vogeli*, *Ehrlichia canis*, *Hepatozoon canis*, *Mycoplasma* spp., Dogs, Cats, Qatar

## Abstract

**Background:**

Vector-borne diseases (VBDs) have been increasingly reported in dogs and cats worldwide. However, no data are currently available regarding canine and feline VBDs in Qatar and limited information is available from other Persian Gulf countries.

**Methods:**

Blood samples from 98 client-owned animals (i.e. 64 dogs and 34 cats) living in Doha (Qatar) were collected and the presence of genomic DNA of *Anaplasma* spp., *Babesia* spp., *Dirofilaria* spp., *Ehrlichia* spp., *Hepatozoon* spp., *Mycoplasma* spp. and *Rickettsia* spp. was assessed by polymerase chain reaction (PCR), real time-PCR (rt-PCR) and sequence analysis.

**Results:**

Of the 64 dogs, 12 (18.8%) were infected with at least one pathogen (i.e. 7.8% with *Mycoplasma* spp., 4.7% with *Babesia vogeli*, 3.1% with *Ehrlichia canis*, and 1.6% with *Anaplasma platys*, *Babesia gibsoni* and *Hepatozoon canis*, each). One of the 12 dogs was co-infected with *B. vogeli* and *E. canis*. Of the 34 cats, seven (20.6%) animals were infected with at least one pathogen (i.e. 5.9% were positive for *Mycoplasma* spp., and 2.9% for *Babesia felis*, *B. vogeli*, *E. canis*, “*Candidatus* Mycoplasma haemominutum” and *Mycoplasma haemofelis*, each). No dogs or cats were positive for *Dirofilaria* spp. or *Rickettsia* spp.

**Conclusions:**

Although the sample sizes of dogs and cats herein analysed was moderately small, data from this study report the occurrence of *A. platys*, *B. vogeli*, *B. gibsoni*, *E. canis*, *H. canis* and *Mycoplasma* spp. in domestic dogs and of *B. felis*, *B. vogeli*, “*Candidatus* M. haemominutum”, *E. canis* and *M. haemofelis* in domestic cats from Qatar. Further investigations along with prophylactic measures are strongly recommended in order to reduce the risk of dogs and cats acquiring VBDs in Qatar.

## Background

Canine and feline vector-borne diseases (VBDs) are caused by a wide range of pathogens, comprising viruses, bacteria, protozoa and helminths, transmitted to dogs and cats by different species of arthropod vector [1]. Some of these pathogens may represent a serious threat to animal health and welfare, and constitute a diagnostic challenge to practitioners due to the wide spectrum of clinical manifestations, the long prepatent periods and the frequent occurrence of co-infections [2]. For instance, pathogens belonging to the genera *Anaplasma*, *Babesia*, *Ehrlichia*, *Hepatozoon* and *Rickettsia* cause tick-borne diseases of major concern in dogs and cats [3–6].

Nowadays, VBDs are increasingly reported in several regions of Europe [7, 8]. Recent studies have reported that demographic and political changes, increased global transport, deforestation, urbanization, abundance of wildlife hosts, climate alterations and the availability of more accurate diagnostic tools may have accounted for this expansion [8–10]. In Europe there are reports of canine VBDs spreading to non-endemic countries; examples include dirofilariosis caused by *Dirofilaria immitis* and *Dirofilaria repens*, which is spreading to northern and Eastern Europe [11], and babesiosis caused by *Babesia canis* which is extending towards northern European countries [12, 13]. Nonetheless, the scant investigation of vector-borne pathogens (VBPs) may underestimate their prevalence and distribution in several countries. This is the case of Qatar, a country located in the northern hemisphere desert, Middle East, bordering Saudi Arabia. In recent years, as a result of massive immigration, Qatar has experienced a rapid demographic growth and a concomitant increase in the number of pets. These factors, along with the documented population’s shift from the rural areas to populated urban centres like Doha, have enhanced the risk of exotic VBDs [14].

As no data are available regarding VBDs and associated pathogens in cats and dogs from Qatar, a comprehensive molecular survey was conducted to assess the occurrence of agents belonging to the genera *Anaplasma*, *Babesia*, *Dirofilaria*, *Ehrlichia*, *Hepatozoon*, *Mycoplasma* and *Rickettsia* in dogs and cats from Doha.

## Methods

### Study location

Qatar has a desert climate with an arid and hot summer characterized by temperatures ranging between 25 and 46 °C, and a relatively mild winter, with minimal rainfall. Doha is the country’s capital and its largest city.

### Animals sampling

From March to July 2016, 98 randomly enrolled owned pets (i.e. 64 dogs and 34 cats) living in Doha city and its outskirts were presented to a veterinary medical centre in Doha for routine procedures (animals were clinically healthy). Overall, 39 male and 25 female dogs, with ages ranging from 2 months to 13 years (median: 24 months) were examined. Almost half were mixed-breeds (*n* = 33; 51.6%) and the other half pure breeds (*n* = 31; 48.4%). Fifty dogs (78.1%) were born in Qatar and had no history of travelling, whereas the remaining (*n* = 14; 21.9%) were imported from a range of countries (e.g. Oman, UK, United Arab Emirates, USA and the Ukraine).

Of the enrolled cats, 23 were males and 11 were females, with ages ranging from 6 months to 13 years (median: 24 months). All cats were from pure breeds (domestic short-haired, domestic long-haired, Persian and Himalayan). Nine cats were exclusively kept indoors and the remaining 25 had outdoor access; 32 were born in Qatar and had no history of travelling abroad, while two were imported, one from South Africa and the other from the Ukraine. Ectoparasites (only tick specimens) were detected in nine animals (*n* = 6 dogs; *n* = 3 cats), although not collected for the present study.

Whole blood was collected from each animal by venipuncture of the cephalic and/or jugular veins. Two drops of whole blood (~80 μl) were spotted onto the centre of two separate filter papers, air-dried for 3–4 h and individually stored in a sterile bag at -20 °C.

### Molecular procedures and pathogens investigated

Genomic DNA was extracted from the filter papers using a commercial kit (QIAampDNA Micro Kit, Qiagen, Hilden, Germany), according to the manufacturer’s instructions. DNA of *Anaplasma* spp., *Ehrlichia* spp., *Hepatozoon* spp. and *Dirofilaria* spp. was detected by conventional PCR (cPCR) using primers targeting partial 16S rRNA, 18S rRNA and cytochrome *c* oxidase subunit 1 (*cox*1) genes (350, 600 and 689 bp, respectively) and protocols described elsewhere [15–17]. DNA of *Mycoplasma* spp. was detected using SYBR Green real-time PCR (rt-PCR) assay by amplification of two fragments for 16S rRNA gene of different size (i.e. 127 and 259 bp) as described elsewhere [18, 19], whilst *Rickettsia* spp. was detected using SYBR Green rt-PCR assay by amplification of *rompB* (489 bp) gene, as previously described [20]. Blood samples were also tested for *Babesia* spp. and *Theileria* spp. (18S rRNA), both by cPCR (410 bp) and rt-PCR (411–452 bp) [21, 22]. For all PCR tests, positive (DNA of a pathogen-positive blood sample) and negative (no DNA) controls were included. PCR products were examined on 2% agarose gels stained with GelRed (VWR International, Milano, Italy) and visualized on a GelLogic 100 gel documentation system (Kodak, New York, USA). The amplicons were purified and sequenced in both directions using the same primers as for PCR, employing the Big Dye Terminator v.3.1 chemistry in a 3130 Genetic Analyzer (Applied Biosystems, Foster City, CA, USA). Sequences were aligned using the ClustalW program [23] and molecular identification achieved by comparison with those available in the GenBank database by the Basic Local Alignment Search Tool (BLAST; http://blast.ncbi.nlm.nih.gov/Blast.cgi).

### Data analysis

Exact binomial 95% confidence intervals (CI) were established for proportions. The Chi-square and Fisher’s exact tests were used to compare proportions, with a probability *P*-value < 0.05 regarded as statistically significant. Analyses were done using the StatLib software.

## Results

Out of the 64 dogs tested, 12 (18.8%) were positive for at least one pathogen. Five (7.8%) dogs were positive for *Mycoplasma* spp. (not identified to the species level by sequencing), three (4.7%) for *Babesia vogeli*, two (3.1%) for *Ehrlichia canis*, and one (1.6%) for *Anaplasma platys*, *Babesia gibsoni* and *Hepatozoon canis*, each. Co-infection with two pathogens (i.e. *B. vogeli+E. canis*) was molecularly detected in one dog (1.6%) (Table 1).

**Table 1:** Prevalence, accession number and percentage of nucleotide identity of vector-borne pathogens detected in 64 dogs from Doha (Qatar)

Pathogen	Positive dogs			Accession number, percentage of nucleotide identity
	*n*	%	95% CI	
Single infections	11	17.2	8.9–28.7	
*Anaplasma platys*	1	1.6	0.0–8.4	KU500914; 100%
*Babesia gibsoni*	1	1.6	0.0–8.4	KP666166; 100%
*Babesia vogeli*	2	3.1	0.4–10.8	KU662366, KT333456; 100%
*Ehrlichia canis*	1	1.6	0.0–8.4	KR920044; 100%
*Hepatozoon canis*	1	1.6	0.0–8.4	KJ605145; 100%
*Mycoplasma* spp.	5	7.8	2.6–17.3	ns
Co-infections	1	1.6	0.0–8.4	
*B. vogeli* + *E. canis*	1	1.6	0.0–8.4	
Single + co-infections (≥ 1 agent)	12	18.8	10.1–30.5	

*Abbreviations*: *95% CI*, 95% confidence interval; *ns*, not sequenced

Seven out of 34 cats (20.6%) were positive for one of the detected vector-borne pathogens. Two cats (5.9%) were positive for *Mycoplasma* spp., and one (2.9%) for *Babesia felis*, *B. vogeli*, *E. canis*, “*Candidatus* Mycoplasma haemominutum” and *Mycoplasma haemofelis*, each (Table 2).

**Table 2:** Prevalence, accession number and percentage of nucleotide identity of vector-borne pathogens detected in 34 cats from Doha (Qatar)

Pathogen	Positive cats			Accession number, percentage of nucleotide identity
	*n*	%	95% CI	
*Babesia felis*	1	2.9	0.1–15.3	AY452707; 99%
*Babesia vogeli*	1	2.9	0.1–15.3	KT333456; 100%
*Ehrlichia canis*	1	2.9	0.1–15.3	KR920044, 100%
*Mycoplasma* spp.	2	5.9	0.7–19.7	ns
*Mycoplasma haemofeli*s	1	2.9	0.1–15.3	KU645929; 99%
“*Candidatus* Mycoplasma haemominutum”	1	2.9	0.1–15.3	KU645935; 99%
Single infections	7	20.6	8.7–37.9	

*Abbreviations*: *95% CI*, 95% confidence interval; *ns*, not sequenced

BLAST analysis confirmed the pathogen identification with the highest nucleotide identity (i.e. 99–100%, Tables 1 and 2) with the sequences available in the GenBank database (accession numbers: AY452707; KJ605145; KP666166; KR920044; KT333456, KU500914; KU645929; KU645935; KU662366). No animal was positive for *Dirofilaria* spp. and *Rickettsia* spp.

No statistically significant associations were found for positivity to VBPs among the categories of gender (*P* = 0.728), juvenile vs adult (*P* = 0.240), pure vs mixed-breeds (*P* = 1.0), born in vs out of Qatar (*P* = 0.437), or indoor vs outdoor lifestyle (*P* = 1.0). All representative sequences obtained are available in the GenBank database under accession numbers: MF140995–MF140999 and MF142765–MF142769.

## Discussion

Data obtained from this study indicate that dogs and cats from Doha city, Qatar, are infected with several VBPs, which have been identified by sequence analysis. Indeed, although the sample sizes of dogs and cats herein analysed was small, the occurrence of *A. platys*, *B. vogeli*, *B. gibsoni*, *E. canis*, *H. canis* and *Mycoplasma* spp. have been recorded in domestic dogs; and of *B. felis*, *B. vogeli*, “*Candidatus* M. haemominutum”, *E. canis* and *M. haemofelis* in domestic cats. Results show that approximately one fifth of the dogs (18.8%) and cats (20.6%) are infected with tick-borne pathogens, taking into account that all the detected agents are vectored or potentially vectored by ticks. This finding is of relevance considering that sampled animals are client-owned pets and may underrepresent the overall population, including stray animals.

Haemotropic mycoplasmas were the most prevalent VBPs detected in this study. Few data are available on the status of hemoplasmas in the Middle East, with one study showing the presence of the three feline species or candidate species (*M. haemofelis*, “*Candidatus* M. haemominutum” and “*Candidatus* Mycoplasma turicensis”) in Iranian cats [24]. In the present study, *M. haemofelis* and “*Candidatus* M. haemominutum” were detected in cats from Qatar, and *Mycoplasma* spp. was found in dogs, thereby expanding the current knowledge of the distribution of feline and canine hemoplasmosis in the Middle East. The occurrence of *A. platys* and *E. canis* in dogs has been previously reported in the Middle East region, specifically in dogs from Turkey [25], and of *Ehrlichia* spp. from Saudi Arabia [26]. Additionally, in Israel and Egypt dogs were found to have antibodies to *E. canis* [27–29], whereas antibodies to *Anaplasma* spp. have been demonstrated only by immunofluorescence antibody tests in domestic dogs [29]. Conversely, canine hepatozoonosis caused by *H. canis* has been reported several times from regions of the Middle East, particularly in Israel [30], Iraq [31] and Egypt [32]. In the present study, three distinct species of *Babesia* were detected in Qatar, namely *B. felis*, *B. gibsoni* and *B. vogeli.* Although the virulence of these intraerythrocytic protozoans varies among species, *B. gibsoni* is known to cause highly severe disease [33] and may represent a serious threat for dogs living in Qatar. *Babesia felis* was here identified in a cat imported from South Africa, where clinical babesiosis has been reported in domestic cats [34]. Except for *B. felis*, all the detected agents were found in pets that had never travelled outside of Qatar, suggesting that these infections were locally acquired and that these pathogens are endemic in Doha.

Most of the detected pathogens are vectored or suspect to be vectored by *Rhipicephalus sanguineus* (*sensu* *lato*), a tick species displaying a worldwide geographical distribution, a vast competency to transmit pathogens and a great adaptability to survive in distinct ecosystems [1, 35]. However, no information is available on its occurrence in Qatar, only in nearby countries such as Saudi Arabia and the United Arab Emirates [26, 36].

The lack of epidemiological studies in the Arabian Peninsula prevents any speculation on the impact of VBPs on the health of dogs and cats. However, the hot and arid climate of Qatar has shown to be potentially suitable to the perpetuation of vectors and transmission of several arthropod-borne diseases. The ascending development of Qatar’s economy has led to an inflow and outflow of people and their pets. This factor may lead to an increase risk of introduction and spreading of VBPs. Therefore, effective prophylaxis, as well as routine screenings should be advocated in pets from Qatar. Diagnostic screenings should also be performed when formulating Pet Travel Schemes of animals relocating to and from the Middle East region.

## Conclusions

This study expands the information on the distribution of canine and feline VBPs in dogs and cats inhabiting Qatar. Further studies, including a larger number of pets and populations from other areas are needed to better understand the epidemiological scenario of VBDs in the country. Considering the impact of such diseases on the health of cats and dogs, routine screenings and prophylactic measures are strongly recommended.

## Abbreviations

PCR: Polymerase chain reaction
rt-PCR: Real-time PCR
VBDs: Vector-borne diseases
VBPs: Vector-borne pathogens
